# Paracentral Acute Middle Maculopathy and Nonischemic Central Retinal Vein Obstruction in a Young Patient with Protein C Deficiency

**DOI:** 10.1155/2022/1237148

**Published:** 2022-06-06

**Authors:** Mojtaba Abrishami, Seyedeh Maryam Hosseini, Nasser Shoeibi, Hamid Reza Heidarzadeh

**Affiliations:** Eye Research Center, Mashhad University of Medical Sciences, Mashhad, Iran

## Abstract

**Purpose:**

To report a case of protein C deficiency with paracentral acute middle maculopathy (PAMM) and nonischemic central retinal vein obstruction (NI-CRVO). *Case Report*. A previously healthy twenty-one-year-old male was referred with sudden-onset, painless, unilateral paracentral scotomata in the right eye for a week. His uncorrected visual acuity was 20/20 in both eyes. In fundus examination of the right eye, mild venous tortuosity, retinal hemorrhages, and a gray-white opacity in the papillomacular bundle were observed. In the macular spectral-domain optical coherence tomography images, a hyperreflective band was noticed at the level of the inner nuclear layer, indicating PAMM. Fundus appearance and fluorescein angiography findings indicated NI-CRVO diagnosis made. The systemic and laboratory evaluations disclosed a protein C deficiency.

**Conclusion:**

In this report, in a healthy young male, combined PAMM and NI-CRVO showed to be the cause of blurred vision in the setting of protein C deficiency.

## 1. Introduction

Paracentral acute middle maculopathy (PAMM) is an ischemic retinal lesion of the inner nuclear layer (INL). It is the result of global hypoperfusion at the level of the retinal capillary plexuses (RCP), especially the deep vascular complex (DVC) [1]. The most common associated conditions with PAMM are retinal vascular diseases like central retinal vein occlusion (CRVO) and central retinal artery occlusion [2]. The majority of CRVO cases are older than 50 years, and it is a rare condition in younger patients [3]. Primary thrombophilia like protein C deficiency is found rarely among patients with CRVO, and its association with this condition is unclear [4].

In this report, we present a young man without any remarkable past medical history who developed PAMM and nonischemic CRVO (NI-CRVO) in the setting of protein C deficiency.

## 2. Case Report

**Figure 1:**
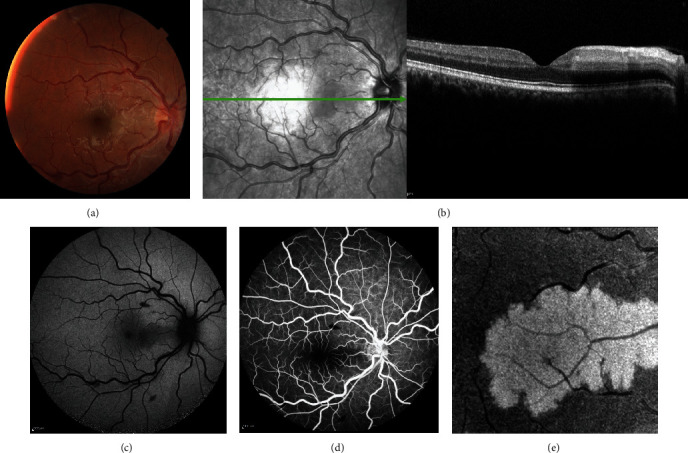
(a) The fundus photograph of the right eye shows disc swelling, multiple dot-like retinal hemorrhages, venous tortuosity, and a gray-white opacity in the papillomacular bundle. (b) Spectral-domain optical coherence tomography illustrates a hyperreflective band in the level of INL, and in infrared reflectance image, a grayish lesion with sharp borders in the papillomacular bundle can be observed. (c) Fundus autofluorescence shows the lesion as a hypoautofluorescence lesion. (d) The late phase of fluorescein angiography (3′ : 1^″^) discloses no significant nonperfusion area. (e) 3∗3 mm en face optical coherence tomography angiography image of the right eye lesion revealed edema in the level of deep capillary plexus.

A twenty-one-year-old male without significant past medical history presented with sudden onset, painless, unilateral blurred vision in the right eye since one week before. Uncorrected visual acuity (UCVA) for both eyes was 20/20. The relative afferent pupillary defect was negative. The slit-lamp examination had no pathologic finding, and intraocular pressure was in the normal range. The fundus examination of the right eye showed disc swelling, multiple diffuse spot-like retinal hemorrhages, venous tortuosity, and a gray-white opacity retinal patch in the papillomacular bundle. The left eye was entirely normal (Figure 1(a)). In macular spectral-domain optical coherence tomography (SD-OCT), a hyperreflective band in the level of INL was noticed (Figure 1(b)). In fundus autofluorescence, the white opacity in the fundus examination was revealed as a hypoautofluorescence lesion (Figure 1(c)). Fluorescein angiography disclosed a slight delay in the venous phase without any significant nonperfusion area in the late phase (Figure 1(d)). Optical coherence tomography angiography images of the retinal lesion area showed flow deficit in the level of the choriocapillaris and a hyperreflective area in the level of the deep capillary plexus (Figure 1(e)).

With a diagnosis of PAMM and NI-CRVO in the right eye, evaluation for systemic diseases and the hypercoagulopathy states was performed. Complete blood count and biochemistry tests, including fasting blood sugar and hemoglobin A1C, were in the normal ranges. He had normal values for prothrombin time (PT), partial thromboplastin time (PTT), international normalized ratio (INR), protein S, factor 5 Leiden, antiphospholipid, and anti-cardiolipin immunoglobulin G. The only significant abnormality in systemic evaluations was a low level of protein C (66.5%). Consultation with a hematologist for further evaluation was done, and the diagnosis of protein C deficiency was confirmed. Regarding the coronavirus 2019 (COVID-19) pandemic, we evaluated COVID-19 infection. Neither nasopharyngeal swab sample for reverse transcriptase polymerase chain reaction test nor antibodies against severe acute respiratory syndrome coronavirus 2 was conclusive for COVID-19.

**Figure 2:**
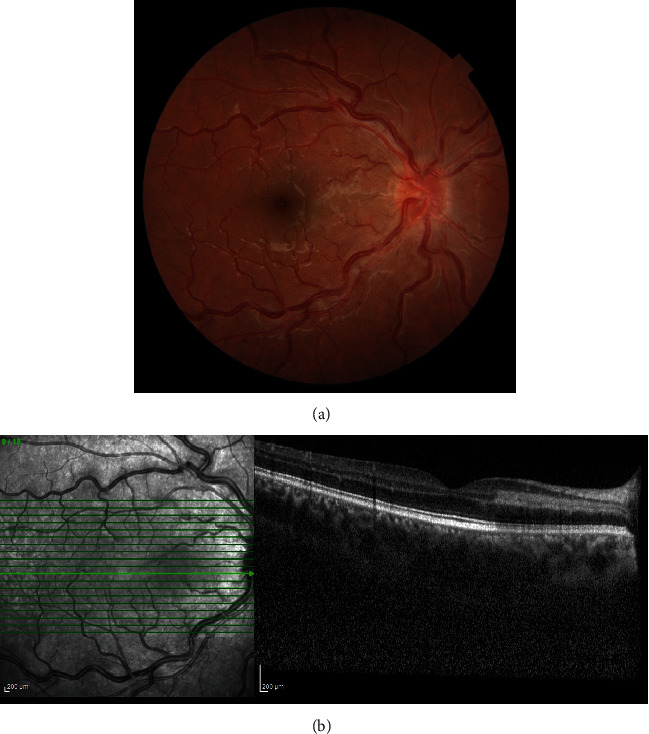
Fundus imaging following one month. (a) Fundus photograph of the right eye shows the vanishing of dot-like retinal hemorrhages and decrease in the opacity of papillomacular bundle lesion. (b) SD-OCT of the right eye revealed diminution in the thickness and edema of the hyperreflective retinal lesion.

Besides the hematology consult, the patient was followed, and after one month, he had a stable vision with no decreased visual acuity. Macular SD-OCT demonstrates decreased thickness and attenuation of the hyperreflective band in INL (Figure 2).

## 3. Discussion

Herein, we report PAMM and NI-CRVO in a young patient with protein C deficiency. CRVO is mainly associated with age, and nearly 90% of cases are older than 50 [3]. The association of systemic disease with CRVO is less prominent in young patients; on the other hand, thrombophilia is more associated with CRVO [5]. Regarding the young age of our patient, evaluation for coagulopathy disorders was performed and resulted in the diagnosis of protein C deficiency.

CRVO is the most common cause of PAMM. A retrospective, observational case-series of 484 American patients with NI-CRVO reported that the prevalence of PAMM development was approximately 5.17% [6]. In a case series of patients with PAMM and retinal vascular disease in Chinese patients, the prevalence of PAMM was 5.23% in 555 patients with retinal vein occlusion. They also reported its prevalence was 19.44% in patients with retinal artery occlusion [7].

Clinical manifestation of PAMM can be unilateral or bilateral and typically presents with one or multiple paracentral scotomata, blurred central vision, and difficulty in focusing. The visual acuity can be normal, like in our case, or slightly decreased. Fundus examination can be normal or associated with deep, smooth grayish lesions [2]. SD-OCT shows PAMM lesions as a hyperreflective band in the INL that progress to atrophy with INL thinning over time, and OCTA shows the extent of ischemia in the inner and deep capillary plexus [8].

PAMM has been reported being associated with systemic diseases like idiopathic intracranial hypertension, meningitis, leptomeningeal infiltration by tumors, carotid arterial disease, vascular surgeries, antiphospholipid syndrome, giant cell arteritis, and some drugs [2]. Ocular disease associations are reported as inflammatory chorioretinopathy, congenital glaucoma, and foveal hypoplasia. Also, it was reported in the otherwise healthy patients following viral flu-like disease, H1N1 vaccination, and pregnancy [2].

Protein C is a critical vitamin K-dependent protease in the regulation of thrombin. It also has some cytoprotective functions in the regulation of inflammation and sepsis. The mild protein C deficiency incident is about 1 in 200 to 1 in 500, but the severe deficit incident is about 1 in 20000 people [9].

Protein C deficiency has been reported in patients with CRVO, but in a large series with control groups, there was no significant association [4]. To the best of our knowledge, there is no report on PAMM association with protein C deficiency; however, in the review of its pathogenesis, it is identified secondary to DVC flow failure, and it could be secondary to thrombosis in the setting of protein C deficiency [10].

Here, we report a previously healthy young male who developed PAMM and NI-CRVO in the setting of protein C deficiency, and it is the first time that a case of protein C deficiency with PAMM has been reported. In conclusion, PAMM and NI-CRVO could develop in young patients. However, the association between primary thrombophilia disorders, like protein C deficiency, and the retinal vessel occlusive disorders is unclear.

## Data Availability

The datasets used during the current study are available from the corresponding author on reasonable request.
